# Phase synchronization modes are associated with heterogeneous social orienting in children and adolescents with autism

**DOI:** 10.1186/s13229-026-00729-w

**Published:** 2026-07-09

**Authors:** Xing-Ke Wang, Sheng Yang, He-Li Lu, Chun-Yan Yue, Li-Fang Dai, Shao-Di Wang, Shuang Li, Zhou Zhang, Li-Rui Hao, Rui-Xuan Ren, Yue Cao, Gaoxiang Ouyang

**Affiliations:** 1 State Key Laboratory of Cognitive Neuroscience and Learning, Beijing Normal University, Beijing, 100875 China; 2 The Second Affiliated Hospital of Nanchang University, Nanchang, 330000 China; 3 Beijing Jianxiang School, Beijing, 100875 China; 4 Department of Neurology, Beijing Children’s Hospital, Capital Medical University, National Center for Children’s Health, Beijing, 100045 China; 5 Peking University Third Hospital, Beijing, 100191 China

**Keywords:** Autism spectrum disorder, Social communication, Heterogeneity, Naturalistic viewing, Eye tracking, EEG, Microstates, Alpha oscillations, Phase

## Abstract

**Background:**

Social deficit in autism spectrum disorder (ASD) varies substantially across individuals, yet the neural mechanisms underlying this variability remain poorly understood. Resting state electrophysiological measures may under-engage social information processing and may be less sensitive to ASD-related neural differences. Here we combined EEG with eye tracking during a low demand viewing paradigm to probe neural dynamics and to identify data-driven neurodynamic modes associated with variability in social orienting.

**Methods:**

We recruited 88 autistic and 71 typically developing (TD) participants for eyes-open resting-state EEG. A subset of these participants, including 58 autistic and 61 TD participants, additionally completed a Social vs. Geometric paradigm with simultaneous EEG and eye tracking. Alpha-band resting-state and task-state EEG were segmented into five microstate (MS) classes (A–E). We compared MS temporal and complexity features between conditions and used support vector machine classification to test whether resting-state or task-state MS features better differentiated ASD from TD participants. For the more discriminative condition, MS-based alpha activity was further characterized by amplitude and phase-locking value (PLV). Participant-level MS-based PLV features were then used for k-means clustering, and moderation models examined whether PLV shaped the association between autistic traits and social orienting.

**Results:**

Task-state MS features differentiated ASD from TD more accurately than resting-state features. Group differences were primarily expressed in MS-based alpha PLV across the five MS classes, whereas alpha amplitude showed no significant group differences. Clustering identified two PLV-based synchronization modes that were present in both ASD and TD participants. Within ASD, these modes differed in social orienting, and MS A PLV moderated the association between autistic traits and social scene preference ratio.

**Limitations:**

Given the cross-sectional design, tracing the developmental trajectories of these distinct neurodynamic modes will require future multi-center, longitudinal tracking.

**Conclusions:**

These findings suggest that social orienting variability within ASD is associated with heterogeneous neurodynamic modes that become most visible under naturalistic social input and are more strongly associated with phase synchronization.

**Supplementary Information:**

The online version contains supplementary material available at https://doi.org/10.1186/s13229-026-00729-w.

## Introduction

Social deficit is a core symptom of autism spectrum disorder (ASD), and it varies substantially across individuals in severity and behavioral expression [1]. Although behavioral instruments such as the Social Responsiveness Scale − 2nd Edition (SRS-2) describe this variability across dimensions of social functioning (e.g., social awareness, social cognition, social communication, social motivation, and autistic mannerisms) [2], many group-level neurobiological studies have treated social deficit as a relatively unitary construct. Such approaches, however, may obscure meaningful inter-individual differences in the neural mechanisms underlying social deficit in ASD. Increasing evidence suggests that ASD is characterized by substantial etiological and neurobiological heterogeneity [3]. At the level of functional connectivity, findings from studies increasingly point to pronounced inter-individual variability rather than a single uniform deficit [4–7]. Together, these observations suggest that social deficit in ASD may reflect multiple neurophysiological modes, including (but not limited to) (i) trait-like anomalies in social brain circuitry that directly constrain social functioning [8] and (ii) differences in neural regulation or compensation that partially offset deficits through alternative strategies or pathways [9]. What remains missing is a mechanistic account of this heterogeneity that describes how large-scale neural coordination reconfigures to support moment-to-moment social information processing.

A major obstacle is that many EEG studies on large-scale network dynamics in ASD under the age of 18 have relied on resting-state recordings. Resting-state EEG provides an important window into intrinsic oscillatory organization, and evidence indicates that resting activity may be related to subsequent task-related responses. For example, reduced resting alpha power in children with ASD has been associated with attenuated alpha desynchronization to behaviorally relevant stimuli [10]. Large-scale oscillatory hypoconnectivity has also been observed in ASD across eyes-open resting-state, attentional-control, and social-cognition task conditions, suggesting that some network-level alterations may generalize across recording states [11]. However, social deficit typically unfolds in dynamic, multimodal contexts [12], and resting-state paradigms impose no explicit demand to sample and integrate external social information. This limitation is important because task-based EEG studies suggest that group differences may emerge in state-dependent modulation even when baseline connectivity does not differ between ASD and typically developing (TD) [13]. These findings indicate that resting-state EEG can characterize intrinsic oscillatory organization, whereas task-related EEG is needed to examine how neural coordination is dynamically modulated by external inputs and behavioral demands.

Naturalistic viewing paradigms combined with eye tracking offer a solution because they provide ecologically meaningful social inputs while simultaneously quantifying individual differences in social orienting. In the Social vs. Geometric paradigm [14–16], participants freely view paired dynamic social and nonsocial stimuli under low cognitive demand, allowing spontaneous attentional allocation to unfold. Evidence from related preferential-looking paradigms indicates that atypical social orienting can be detected early in ASD [17]. In social versus geometric viewing, toddlers with ASD spend more time fixating on dynamic geometric images, and a geometric fixation proportion above 69% has shown high positive predictive value for ASD classification [16]. Subsequent work further suggests that enhanced preference for geometric repetition may characterize an ASD subgroup with greater clinical severity [18]. Consistent with this interpretation, ASD toddlers classified within the geometric preference subtype showed greater symptom severity and poorer cognitive and adaptive functioning [14]. Such a naturalistic context may therefore better reveal ASD-relevant neural dynamics tied to social orienting than conventional resting-state recordings.

This leaves an unresolved question of direct relevance to social deficit: whether neural dynamics measured during a stimulus-driven viewing condition provide stronger differentiation between ASD and TD than dynamics measured during unconstrained rest, and whether they better relate to measurable variation in social orienting. To capture neural coordination in this context, we employed EEG microstate (MS) analysis as a unique subsecond window for interrogating these mechanisms [19]. MS is conventionally identified by clustering scalp voltage topographies at peaks of global field power (GFP) in broadband EEG, typically yielding four to five canonical classes (A-E) [20–22]. And these MS topographies and transitions are closely related to alpha-band dynamics [23–25], motivating a mechanistic decomposition of alpha activity during network-state expression. Alpha-band MS studies in ASD demonstrate altered temporal dynamics, including changes in duration, occurrence, and coverage [26, 27]. Additionally, prior work indicates that the canonical topographies of these network states are broadly preserved across conditions [20, 21, 28], whereas their temporal expression is modulated by cognitive and perceptual engagement [29–32]. This property offers a standardized metric to directly contrast intrinsic neural stability with responsiveness to social cues, thereby isolating condition-specific dynamic alterations.

The present study combined EEG and eye tracking to test whether task-related alpha-band MS dynamics during viewing provide information about ASD-related social deficit beyond resting-state measures. We pursued three linked aims. First, we compared resting-state and task-related MS features to determine whether task-related neural dynamics better differentiated ASD from TD participants. We hypothesized that task-derived features would show stronger group discrimination than resting-state features. Second, we examined whether ASD-related MS alterations during task were more closely associated with alpha-band amplitude or phase synchronization. We hypothesized that these alterations would be better captured by MS-specific phase synchronization than by amplitude measures. Third, we tested whether data-driven synchronization profiles moderated the association between autistic traits and social orienting behavior. We hypothesized that distinct synchronization profiles would be associated with different trait-behavior relationships. Together, these analyses were designed to clarify how task-related neural dynamics are related to social orienting heterogeneity in ASD.

## Materials and methods

### Participants and diagnostic assessment

A total of 159 participants were recruited, comprising 88 children and adolescents with ASD and 71 TD controls. All participants were under 18 years of age and were right-handed. Participants were excluded from the task-state analyses due to one of the following reasons: (i) poor visual fixation or task non-compliance (e.g., insufficient valid gaze data below 50% task duration [16, 33], excluded 14 ASD and 4 TD); (ii) excessive task-induced motor artifacts (e.g., head movements during cognitive load, excluded 10 ASD and 5 TD); and (iii) fatigue or refusal to complete the task after the resting-state session (excluded 6 ASD and 1 TD). Consequently, the final analytical sample for the task condition comprised 58 ASD and 61 TD participants. Mean age and detailed demographic and clinical characteristics are summarized in Table 1. Parents or legal guardians provided written informed consent for participation, and children gave verbal assent when developmentally and communicatively able. For children unable to provide verbal assent, participation relied on parental or legal guardian consent, with signs of distress or refusal monitored throughout (e.g. head-shaking, removing the EEG cap). Testing was paused or discontinued if a child showed clear verbal or nonverbal dissent. The study protocol was approved by the Institutional Ethics Committee of the Second Affiliated Hospital of Nanchang University (approval No. I-2025-125) and was conducted in accordance with the Declaration of Helsinki.

**Table 1:** Demographics and clinical characteristics of the participants

	Rest		
	ASD *n* = 88	TD *n* = 71	*p*-value
Age, years, mean (SD)	10.60 (4.49)	10.41 (3.64)	0.77^a^
Female, n (%)	10 (11.36)	29 (47.54)	< 0.001^b^
SRS-2 total (SD)	97.44 (19.78)	40.85 (22.71)	< 0.001^a^

	Tas		
	ASD *n* = 58	TD *n* = 61	*p*-value
Age, years, mean (SD)	9.70 (4.70)	10.16 (3.73)	0.55^a^
Female, n (%)	9 (15.52)	26 (42.62)	< 0.001^b^
SRS-2 total, mean (SD)	87.32 (20.72)	48.28 (14.71)	< 0.001^a^
SSPR (%)	44.40 (12.10)	49.17 (19.81)	0.12^a^

a, two-sample t-tests. b, χ^2^ test

ASD, autism spectrum disorder; TD, typically developing; SRS-2, Social Responsiveness Scale-2nd Edition; SSPR, social scene preference ratio

Participants in the ASD group were recruited from local hospital and had received a prior clinical diagnosis of autism, which was confirmed by experienced clinicians in specialized child mental health services. Exclusion criteria for the ASD group comprised a history of other major neurological disease or brain surgery. Regarding psychotropic medication, participants were required to be medication-free or to have maintained a stable regimen for at least six months prior to the study. TD participants were recruited from local schools and the community. Inclusion criteria required typical developmental history and school placement, with no past or current diagnosis of developmental, psychiatric, or neurological disorders, no regular use of psychotropic medication, and no history of learning difficulties. All included participants had normal or corrected-to-normal vision and hearing according to parental report. Socially related autistic traits were assessed using the caregiver-rated SRS-2. Participants with visible signs of drowsiness or sleep during EEG recording were excluded based on visual inspection.

### Experimental paradigm and behavioral measures

**Fig. 1:**
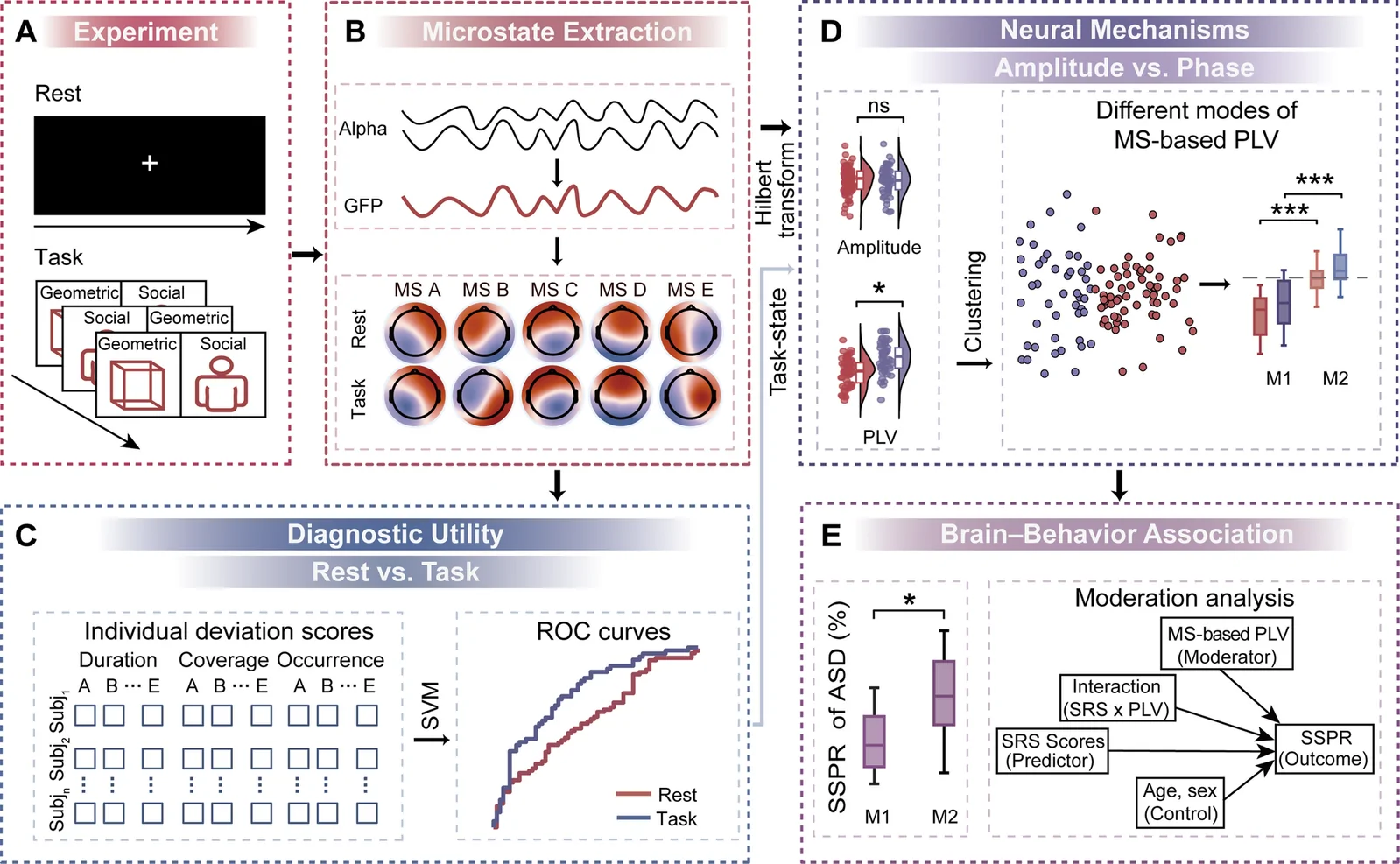
Overview of the experimental design and data analysis. (**A**) Experimental paradigm. The study consisted of two sessions: a resting-state EEG recording (top panel) and a passive viewing task (bottom panel). (**B**) MS extraction procedure. EEG signals were filtered into the alpha frequency band. MS analysis was then performed independently for the resting-state and task-based datasets to identify the five stable MS classes. (**C**) Comparison of the discriminative power of resting-state versus task-based MS in distinguishing ASD from TD. MS features were benchmarked against an age-matched TD reference group to compute individual deviation scores. These deviation scores served as input features for the SVM classification. (**D**) Investigation of neural mechanisms. Focusing on the task-state data, MS-based alpha amplitude and PLV were computed via the Hilbert transform. Unsupervised clustering was performed on the PLV deviation profiles to identify distinct modes. (**E**) Brain-behavior association model. The behavioral and eye-tracking characteristics of the two identified PLV-based modes were first analyzed. Subsequently, a moderation analysis was constructed to test whether PLV modulates the relationship between autistic traits and social visual preference. ASD, autism spectrum disorder; TD, typically developing; EEG, electroencephalography; GFP, global field power; MS, microstate; ns, no significance; PLV, phase locking value; ROC, receiver operating characteristic; M1, mode 1; M2, mode 2; SRS, Social Responsiveness Scale; SSPR, social scene preference ratio; Sub, subject; SVM, support vector machine

Data acquisition comprised two experimental sessions: a resting-state EEG recording and a passive viewing eye-tracking task (Fig. 1A). In both sessions, participants were seated comfortably in a dimly lit, sound-attenuated room, approximately 60 cm from the computer monitor. For the resting-state session, continuous EEG data were recorded for 2 min. Participants were instructed to keep their eyes open, remain as still as possible, and fixate on a cross displayed at the center of the screen to minimize eye movements and muscle artifacts.

The separate passive viewing session was designed to evaluate spontaneous visual preference for social versus non-social stimuli. This task recorded EEG and eye tracking simultaneously. The Social vs. Geometric paradigm, adapted from Moore and colleagues [14] and from the social interest paradigm used in previous studies of neurodevelopmental disorders [15, 34], presented two types of video stimuli simultaneously on a split-screen display. One side of the screen displayed social videos featuring children engaging in naturalistic playful interactions (e.g., dancing or playing games). The other side displayed geometric videos featuring dynamic, colorful geometric shapes (e.g., moving fractals or abstract patterns) with no human figures or social cues. The total duration of the task was 2 min. The positions of the social and geometric videos were swapped every 15 s to control for potential spatial bias. Gaze behavior was recorded using a Tobii Pro X3-120 eye tracker (Tobii Pro, Danderyd, Sweden; https://www.tobiipro.com/product-listing/tobii-pro-x3-120/) at a sampling rate of 60 Hz. Prior to the task, a 5-point calibration procedure was performed to ensure tracking accuracy.

For behavioral analysis, two primary areas of interest were defined corresponding to the social and geometric video regions. The primary behavioral measure was the social scene preference ratio (SSPR), calculated as the percentage of total fixation time spent viewing the social AOI relative to the total fixation time on both areas of interest:

1 $$\:\mathrm{S}\mathrm{S}\mathrm{P}\mathrm{R}=\frac{{T}_{Social}}{{T}_{Social}+{T}_{Geometric}}\times\:100\%$$

A score greater than 50% indicates a preference for social stimuli, while a score lower than 50% indicates a preference for geometric patterns. To characterize social preference across task halves, SSPR was also calculated separately for the first and last halves of the task.

### EEG acquisition and preprocessing

EEG signals were recorded using a 32-channel amplifier. Electrodes were positioned according to the standard 10–20 electrode configuration. The raw data were acquired at a sampling rate of 2000 Hz. Electrode impedances were maintained below 10 kΩ throughout the experiment.

EEG data preprocessing was performed using EEGLAB [35] implemented in MATLAB (R2024a; The Mathworks, USA). Briefly, the raw data were downsampled to 500 Hz to reduce computational load while preserving spectral information. The data were subsequently filtered using a finite impulse response band-pass filter ranging from 1 to 40 Hz to remove slow drifts and high-frequency noise. Malfunctioning or noisy channels were identified and interpolated using spherical spline interpolation. Independent component analysis was performed on the broad-band preprocessed data. The data were then re-referenced to the average reference [36]. Finally, the signals were filtered into alpha (8–13 Hz) frequency for MS analysis [37]. This selection procedure was applied consistently across both the resting-state and task conditions.

### MS segmentation and temporal metrics

#### MS extraction and standard temporal parameters

In line with previously published methodologies utilizing the Microstate Toolbox for EEGLAB [38], MS analysis was performed independently for the resting-state and task-based datasets (Fig. 1B). To justify the number of microstate classes, validation analyses comparing K = 3 to K = 6 solutions were conducted and supported K = 5 as the optimal solution (Table S1 and Fig. S1 in Additional file 1). A standard two-step clustering procedure was then applied to identify five prototypical MS maps (K = 5) for the entire cohort. The clustering algorithm was applied at the points of the local maxima of the GFP, as these time points represent the highest signal-to-noise ratio and stable field configurations. For the initial extraction, selected GFP peaks from each participant were submitted to an atomize and agglomerate hierarchical clustering algorithm to generate individual template maps, disregarding map polarity. To derive group-level representations common across the ASD and TD cohorts, the individual cluster maps from all participants were concatenated and clustered again to compute five global template maps for each condition. The identified global MS classes were then fitted back to the continuous EEG data of each participant to extract MS statistics. Utilizing a winner-takes-all competitive assignment strategy, each time point was labeled with the MS class exhibiting the highest spatial correlation with the momentary scalp topography. The smoothing parameter lambda was set to 0.3 to ensure temporal continuity and minimize noise-induced transitions.

From the fitted MS sequences, we extracted data on three standard temporal metrics. Mean duration represents the average length of time a MS class remains stable before transitioning. Occurrence quantifies the average number of times a MS class appears per second. Coverage indicates the percentage of total recording time occupied by a specific MS class.

#### Computation of individual deviation scores

We calculated deviation scores for each participant (i.e. individual deviation scores) by benchmarking them against an age-matched TD reference group (Fig. 1C). First, to control for sex-related confounding effects, we residualized all 15 MS features (duration, occurrence, and coverage for MS A-E; *j* = 1, …,15) with respect to sex using a general linear model. Specifically, for each participant 𝑖 and feature 𝑗, $\:{x}_{ij}$ denotes the raw MS metric; age- and sex-adjusted values $\:{x^\prime}_{ij}$ were obtained by regressing $\:{x}_{ij}$ on age and sex and using the residuals as inputs for subsequent analyses.

Subsequently, for each ASD and TD participant, we defined a local reference group consisting of the 10 nearest age-matched TD neighbors. A modified z-score was calculated for each feature to quantify the degree of deviation from this age-matched baseline. We used the median and median absolute deviation (MAD) to minimize the influence of outliers:

2 $$\:{Z}_{ij}=\frac{x^\prime_{ij}-M}{c \cdot\mathrm{D}}$$

where $\:{x^\prime}_{ij}$ is the sex-adjusted features for participant *i* and feature *j*, *M* represents the median of the age-matched TD neighbors, and D represents the MAD of the age-matched TD neighbors. The constant c set to 1.4826 is a scaling factor used to ensure that the MAD is asymptotically consistent with the standard deviation for normally distributed data.

Statistical differences in MS dynamics were evaluated using a two-way analysis of variance (ANOVA). The statistical model included group (ASD, TD) and condition (rest, task) as the primary factors. The analysis included individual deviation scores calculated for the mean duration, occurrence, and coverage of all five MS classes. We assessed the main effects of each factor as well as the group-by-condition interaction effect for all dependent variables. Significant interaction effects were further investigated using post-hoc t-tests to identify specific group or condition differences. The Benjamini-Hochberg false discovery rate (FDR) correction was implemented. Statistical significance was established at an adjusted *p*-value threshold of 0.05.

#### Complexity analysis of MS sequences

The non-linear temporal dynamics and information content of the MS syntax were characterized using multiscale sample entropy (MSE). These metrics were computed independently for each participant across both the resting-state and task conditions. The calculation followed a sequence digitization method consistent with previous studies. The symbolic MS classes (A, B, C, D, E) were first converted into a discrete numerical time series (1, 2, 3, 4, 5).

The complexity of the MS time series was quantified using MSE [39], which evaluates predictability across multiple temporal scales to better capture biological signal dynamics. Higher MSE reflects greater complexity and lower predictability, and vice versa. In the present study, MSE was calculated over scales from 1 to 10. The calculation parameters were set to a template length of *m* = 2 and a tolerance threshold of *r* = 0.25 times the standard deviation of the original time series. To capture dynamics at different time resolutions, consecutive coarse-grained time series were constructed by averaging the data points within non-overlapping windows of increasing length (scale factor *τ*). For each scale, a new time series was obtained. Sample entropy was subsequently computed for each coarse-grained time series using the algorithm proposed by Richman [40]. Sample entropy measures the negative natural logarithm of the conditional probability that two sequences of length m (template length) that match within a tolerance threshold r will also continue to match at length *m + 1*.

### SVM classification based on MS individual deviations

To determine whether the resting-state or the task-based condition contains more discriminative information for distinguishing ASD from TD individuals, a linear kernel Support vector machine (SVM) was employed to classify participants as either ASD or TD (Fig. 1C). The input features consisted of individual deviation scores calculated for the mean duration, occurrence, and coverage of all five MS classes.

To ensure the generalizability of the model, we performed a stratified 5-fold cross-validation. In this procedure, the dataset was randomly partitioned into five equal subsamples, with the proportion of ASD and TD participants preserved in each fold. The model was trained on four folds and tested on the remaining one, with the process repeated five times. The classification performance was evaluated using accuracy, sensitivity (correct identification of ASD), specificity (correct identification of TD), and the area under the receiver operating characteristic curve (AUC). This classification pipeline was executed independently for the resting-state and task-based datasets to compare the discriminative power of MS dynamics under different experimental conditions. The SVM feature weights were also examined to identify the most discriminative neurophysiological patterns. In the linear SVM architecture (where ASD is coded as the positive class), a positive weight indicates that higher deviation scores predict an ASD diagnosis, whereas a negative weight indicates that lower deviation scores predict ASD.

### MS-based alpha amplitude and phase locking

To investigate the electrophysiological underpinnings of MS dynamics, we focused on the condition that showed stronger ASD-TD separability in Section “SVM classification based on MS individual deviations” and analyzed the amplitude and phase synchronization of alpha oscillations (8–13 Hz) specific to each MS class (Fig. 1D).

To separate the amplitude and phase dynamics of alpha oscillations, the Hilbert transform was applied to the band-pass filtered EEG signals. The $\:\alpha\:\left(r,t\right)$ denotes the filtered alpha oscillation at electrode *r* and time *t*. The corresponding analytic signal $\:z\left(r,t\right)$ was constructed as:

$$\:z\left(r,t\right)=\alpha\:\left(r,t\right)+i\stackrel{\sim}{\alpha\:}\left(r,t\right)$$

3 $$\:=A\left(r,t\right){e}^{i\phi\:(r,t)}$$

where $\:\alpha\:\left(r,t\right)$ is the Hilbert transform of the real-valued signal, $\:A\left(r,t\right){e}^{i\phi\:(r,t)}$ denotes the instantaneous analytic amplitude (envelope), and $\:\phi\:(r,t)$ represents the instantaneous analytic phase in the interval [*-π*, *+π*].

MS-based amplitude was derived by superimposing the MS label sequence (obtained in Section “MS segmentation and temporal metrics”) onto the continuous analytic amplitude time series. For each MS class (A-E), we extracted the instantaneous amplitude values $\:A\left(r,t\right)$ from all time points where that specific MS was active. These values were then averaged to obtain a mean alpha amplitude for each MS class. These values were compared using independent-samples t tests between the ASD and TD groups.

Phase synchronization was assessed using the phase locking value (PLV), which captures the stability of the phase difference between signals. Similar to the amplitude analysis, the continuous phase time series was segmented based on the MS labels. For any pair of electrodes *j* and *m*, the PLV within MS 𝑘 was computed using only the time indices corresponding to the occurrence of that class:

4 $$\:{PLV}_{jm}^{\left(k\right)}=\left|\frac{1}{{N}_{k}}\sum\:_{\boldsymbol{t}\in\:{\boldsymbol{T}}_{\boldsymbol{k}}}{e}^{i({\phi\:}_{j}\left(t\right)-{\phi\:}_{m}\left(t\right))}\right|$$

where $\:{\boldsymbol{T}}_{\boldsymbol{k}}$ denotes the set of time points assigned to MS *k*, and $\:{N}_{k}$ is the total number of such time points. To obtain a MS-based global index of phase synchrony, we then averaged $\:{PLV}_{jm}^{\left(k\right)}$ across all unique electrode pairs (𝑖, 𝑗), 𝑖<𝑗, yielding a single PLV value for each MS and participant. These values were compared using independent-samples t tests between the ASD and TD groups.

To assess whether MS-specific PLV estimates were influenced by the amount of data entering each estimate, independent-samples t tests compared log-transformed MS-specific EEG time-sample counts between ASD and TD, and Spearman’s correlations tested associations between PLV and log-transformed time-sample counts. In addition, an equal-sample subsampling analysis was performed. For each MS, the minimum number of EEG time samples available across participants was used as the common sampling size. This number of time samples was randomly drawn from each participant within the corresponding MS, and PLV was recomputed across 1000 subsampling iterations. The resulting PLV values were averaged across iterations and used for group comparisons.

To further test whether differences between ASD and TD in amplitude or PLV were specifically linked to MS related dynamics, we examined their association within the ASD group using TD referenced deviation scores. For each participant with ASD, we computed a deviation score by subtracting the TD group mean from the participant value for GFP and for PLV (or amplitude) within each MS. We then estimated the correlation between the deviation of GFP and the deviation of PLV (or amplitude) across ASD participants for each MS. To assess whether this association depended on phase structure, we repeated the same procedure after phase shuffling and recomputed PLV (or amplitude) scores and the corresponding correlations.

### Identification of PLV modes across MS

To identify distinct modes characterizing the heterogeneity of the ASD and TD populations, we performed an unsupervised clustering analysis based on individual deviations in alpha phase synchronization (Fig. 1D).

Following the identical procedure described in Section “MS-based alpha amplitude and phase locking”, we quantified the degree to which each participant’s MS-based PLV deviated from the typical developmental trajectory. Briefly, the PLV values for each of the five MS classes were first adjusted for age and sex related variance. Subsequently, individual deviation scores of PLV (i.e. PLV deviation) were computed by benchmarking each participant against a local reference group of the 10 nearest age-matched TD neighbors. Consistent with the previous classification analysis, we utilized the modified z-score to quantify the deviation from the age-matched baseline. This yielded a 5-dimensional feature vector for each participant, representing their individual deviation profile across the five MS classes.

The five-dimensional PLV deviation profiles were first reduced using principal component analysis, retaining components explaining at least 85% of the variance. k-means clustering was then applied to the retained component scores. The number of clusters was selected based on the consensus of the Silhouette coefficient, Calinski-Harabasz index, Davies-Bouldin index, and Gap statistic across K values (the number of clusters) from 2 to 6. For the chosen K, the stability of the clustering solution was assessed by repeating the k-means algorithm 500 times and calculating the adjusted rand index between the optimal solution and each replication. Finally, we examined the composition of each mode and compared the age distribution across modes using Kruskal-Wallis tests. PLV modes differences were evaluated using a two-way ANOVA.

### Mode comparison of MS-based PLV networks

To examine whether the two ASD modes differed in phase synchronization, we restricted the analysis to MS that showed significant ASD versus TD differences in Section “Identification of PLV modes across MS”. For each selected MS, PLV matrices were extracted. PLV values were residualized with respect to age and sex for each edge within each selected MS. Residualization was performed by fitting a linear regression model to PLV values pooled across the participants included in the comparison, and retaining residuals as residualized PLV. Mode-related differences were then tested edge wise using Welch two sample t tests on residualized PLV values. Effect sizes were summarized using Cohen’s *d*. Edge wise results were mapped back to matrix form for visualization.

For visualization and region level interpretation, electrodes were grouped into six regions and ordered accordingly. The prefrontal electrodes included Fp1 and Fp2. The frontal electrodes included F3, F4, F7, F8, Fz, FC1, FC2, FC5, and FC6. The central electrodes included C3, C4, Cz, CP1, CP2, CP5, and CP6. The parietal electrodes included P3, P4, P7, P8, and Pz. The occipital electrodes included O1 and O2. The temporal electrodes included T7, T8, TP9, TP10, FT9, and FT10.

### Brain-behavior association

#### Correlation analysis

To examine brain-behavior relationships, we tested correlations between MS-based PLV deviation and age and SRS-2 scores within each ASD mode. Correlations were estimated separately within mode 1 and mode 2. The same analysis was conducted in the TD group as a comparison. *p* values were corrected across MS using the FDR.

#### Moderation analyses linking phase synchrony, autistic traits, and social gaze

To further explore the functional and developmental relevance of the identified PLV-based modes, we conducted a moderation analysis to examine whether individual differences in alpha phase synchrony modulate the relationship between autistic trait severity and social visual preference (Fig. 1E). This analysis included both ASD and TD participants. The analysis incorporated four key variables: the SSPR derived from the eye-tracking task as the dependent variable; the total score from the SRS-2 as the independent variable reflecting autistic trait severity; the MS-based PLV from the task condition as the moderator, and age and sex as covariates.

A multiple linear regression model was constructed to test the moderation hypothesis. The SSPR was entered as the outcome variable, with SRS-2 scores and PLV entered as predictors. The model specified the main effects of SRS-2 and PLV, their interaction term ($\:SRS\times\:PLV$), and included standardized age and sex to control for residual developmental effects:

$$\begin{aligned}\:SSPR=&{\beta\:}_{0}+{\beta\:}_{1}\cdot SRS+{\beta\:}_{2}\cdot PLV\cr&+{\beta\:}_{3}\cdot\left(SRS\times\:PLV\right)\cr&+{\beta\:}_{4}\cdot Age+{\beta\:}_{5}\cdot Sex+\epsilon\end{aligned}$$

To decompose significant interaction effects, we performed a simple slope analysis. The relationship between SRS-2 scores and SSPR was estimated at two distinct PLV levels corresponding to the two modes defined in Section “Identification of PLV modes across MS”. The significance of the slope at each level was assessed using t-tests. This analysis allowed us to determine whether the predictive effect of autistic traits on social preference was conditional on the strength of global phase synchronization. All statistical analyses were performed using R.

### Sensitivity analysis

To address the possibility that TD-referenced individualized deviation scores could introduce non-independence between feature normalization and subsequent ASD-TD comparisons, we repeated the key analyses using conventional measures without constructing individualized deviation scores from local TD reference groups.

## Results

### MS segmentation

**Fig. 2:**
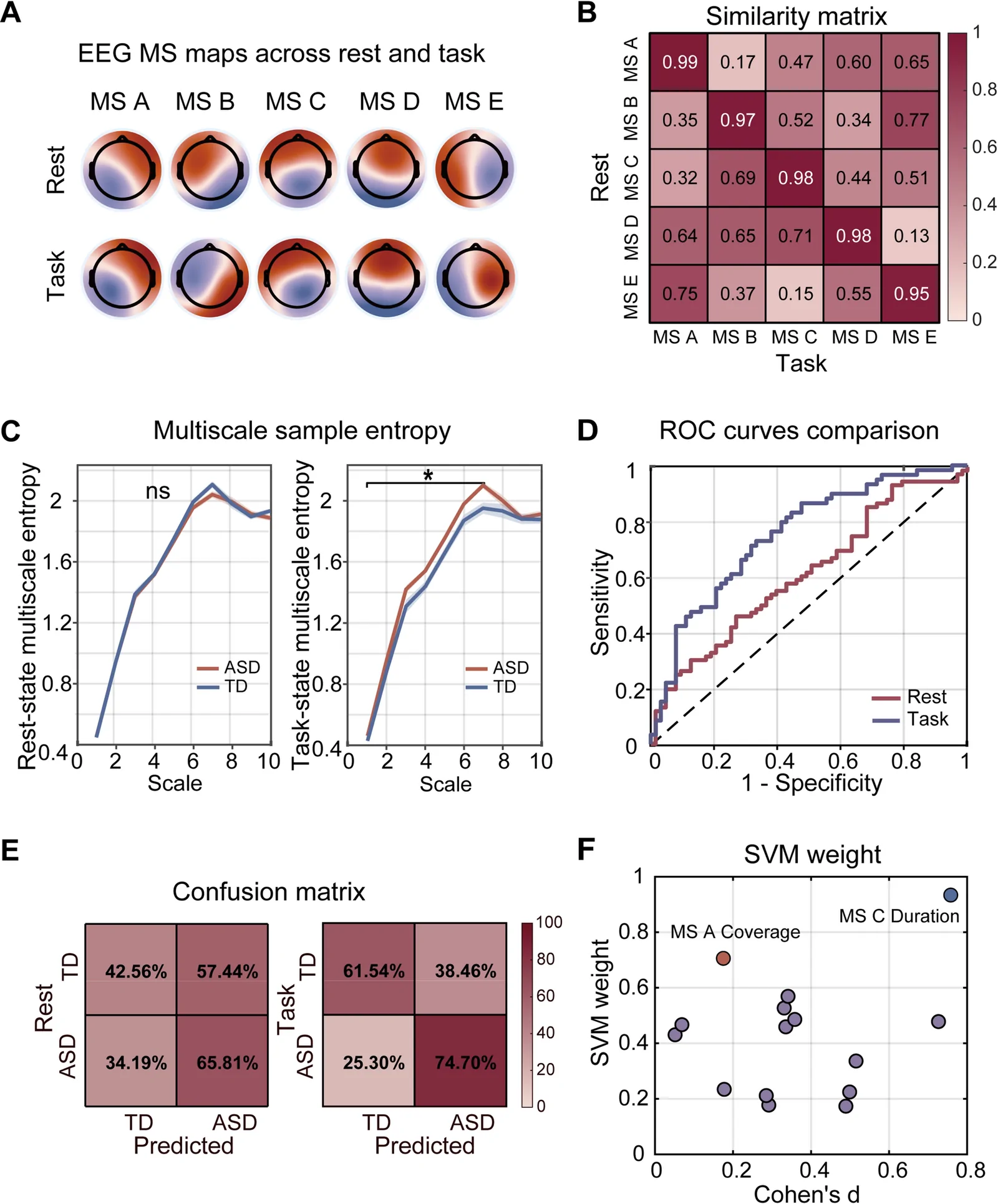
Spatiotemporal characteristics of EEG MS and diagnostic classification performance. (**A**) Topographies of the five MS classes (**A-E**) identified across all participants for the resting state (top panel) and the task (bottom panel). (**B**) Spatial consistency between conditions. The correlation matrix illustrates the high topographic similarity between MS maps derived from the resting-state (y-axis) and the task-based condition (x-axis). (**C**) Complexity analysis using MSE. Shaded areas indicate standard error. (**D**) ROC curves comparing classifier performance for rest (red) and task (blue). (**E**) Classification metrics. Bar graphs show the accuracy, sensitivity, and specificity for both conditions. The task-based model demonstrated superior discriminative power. (**F**) Feature contribution weights. Each point represents one MS feature. MS A coverage and MS C duration are highlighted, reflecting their relatively large group differences and high contribution to the classifier. ASD, autism spectrum disorder; EEG, electroencephalography; MS, microstate; TD, typically developing; SVM, support vector machine; ROC, receiver operating characteristic; *p < 0.05; **p < 0.01; ***p < 0.001; ns, not significant

Preprocessing and MS analysis of the EEG data revealed five MS (labeled A, B, C, D, and E) that best explained the global variance of the dataset (Fig. 2A). The identified MS topographies exhibited distinct spatial distributions consistent with previous literature [24, 41]. The spatial correlation analysis confirmed a high degree of topographic similarity for paired MS across conditions. Specifically, the spatial correlation coefficients between the corresponding resting-state and task-based maps were consistently high: *r* = 0.99 for MS A, *r* = 0.97 for MS B, *r* = 0.98 for MS C, *r* = 0.98 for MS D, and *r* = 0.95 for MS E (Fig. 2B). In contrast, the spatial correlation across different MS was substantially lower, indicating that the five MS represented distinct and stable neural configurations regardless of the experimental condition.

Group differences between participants with ASD and TD were evident across several MS temporal dynamics measures, while condition effects between task and rest were restricted and an interaction was detected for MS C occurrence (full statistics and Fig. S2 are reported in Additional file 1).

### Complexity analysis of MS sequences

The MSE analysis showed that, during the task condition, participants with ASD showed higher MSE than TD participants at scales 1–7 (all FDR-corrected *p* < 0.05), whereas no group difference survived correction in the resting-state condition. Comparisons between resting-state and task conditions further showed that TD participants exhibited lower MSE during the task condition than during resting state at scales 4–7 (all FDR-corrected *p* < 0.05), whereas no condition effect survived correction in the ASD group (Fig. 2C; Tables S2-S3 in Additional file 1).

### SVM classification based on MS individual deviations

To determine whether the resting-state or the task-based condition contains more discriminative information for distinguishing ASD from TD individuals, we implemented a SVM classification framework. The classifier trained on resting-state deviations yielded limited diagnostic utility, achieving a mean accuracy of 55.86% ± 8.23% and an AUC of 0.58 (Fig. 2D). The sensitivity (correct identification of ASD) was 65.81%, and the specificity (correct identification of TD) was 42.56%, indicating that resting-state MS deviations alone were insufficient to reliably distinguish ASD from TD individuals (Fig. 2E). Conversely, the classifier based on task-based deviations demonstrated significantly improved performance, achieving a mean accuracy of 68.03% ± 9.44% and an AUC of 0.75 (Fig. 2D). The model achieved 74.70% sensitivity and 61.54% specificity, suggesting that the task effectively enhanced discriminative signals in MS dynamics (Fig. 2E).

For the task-based model, we examined the SVM feature weights to identify the most discriminative neurophysiological patterns (Fig. 2F). The analysis revealed that the deviation in the coverage of MS A carried the highest positive weight (W = 0.71), indicating that increased coverage of MS A is a strong predictor of ASD. In contrast, the deviation in the duration of MS C carried the highest negative weight (W = -0.93), suggesting that reduced stability of MS C is predictive of ASD.

### MS-based alpha amplitude and phase locking

**Fig. 3:**
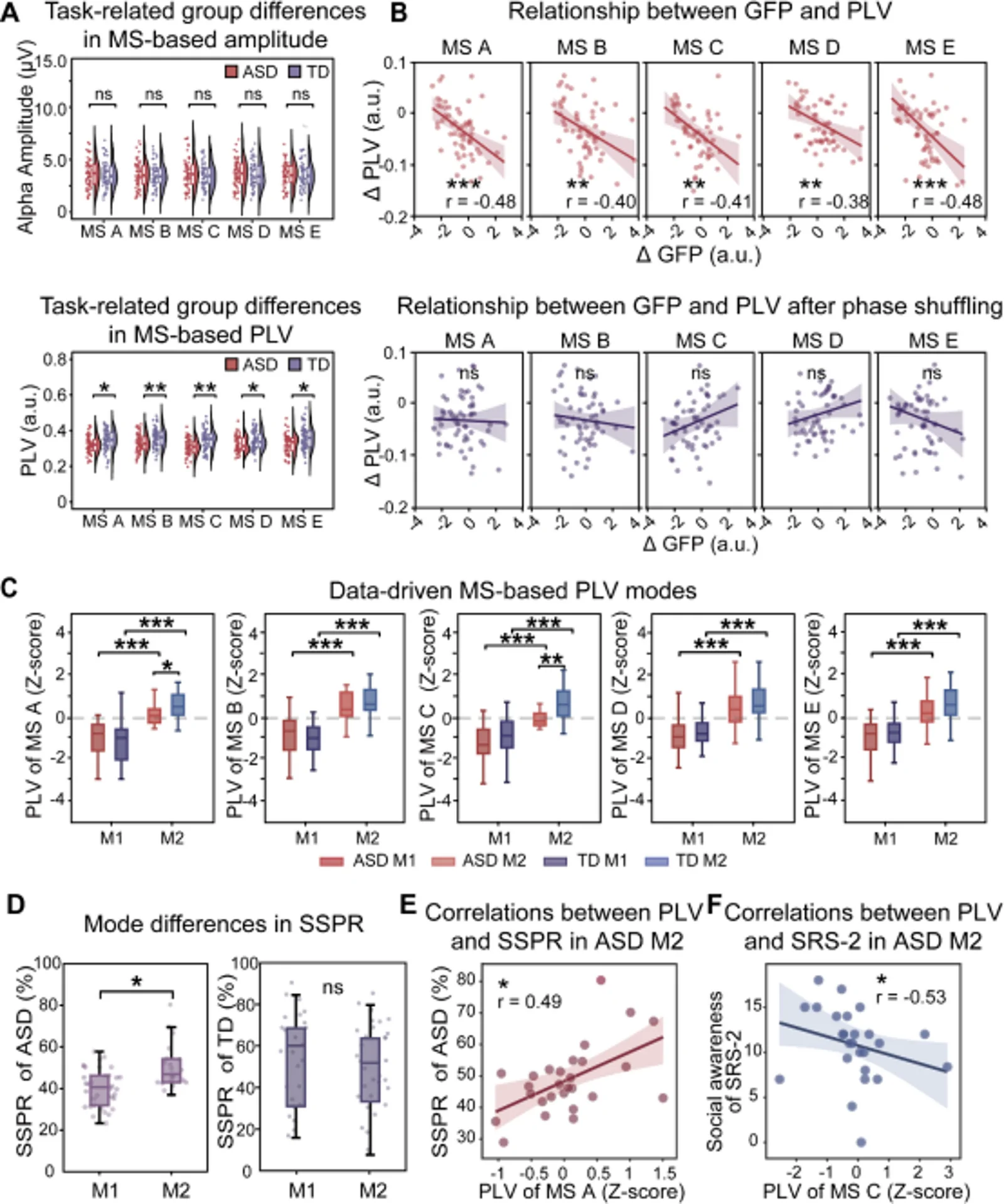
MS-based alpha phase synchronization and identification of modes. (**A**) Group comparisons of alpha amplitude (top panel) and PLV (bottom panel) for each MS. While amplitude showed no significant differences, PLV was significantly reduced in the ASD group across all MS. (**B**) Relationship between GFP and PLV for each MS during the task. Scatter plots show the negative correlation between TD-referenced deviations in GFP and PLV within the ASD group (top panel). This association was abolished after phase shuffling (bottom panel). (**C**) Data-driven PLV modes derived from MS-based PLV features. (**D**) Differences in SSPR between the two PLV modes. Mode 1 exhibited a significantly lower SSPR compared to mode 2 in ASD. (**E**) Correlation between PLV of MS A and SSPR in ASD participants within mode 2. (**F**) Correlation between PLV of MS C and the social awareness subscale of the SRS-2 in ASD participants within mode 2. ASD, autism spectrum disorder; GFP, global field power; MS, microstate; ns, not significant; PLV, phase locking value; M1, mode 1; M2, mode 2; SRS, Social Responsiveness Scale; SSPR, social scene preference ratio; TD, typically developing; *p < 0.05; **p < 0.01; ***p < 0.001

We investigated whether the distinct ASD-TD differences in each MS were driven by underlying variations in oscillatory power or phase synchronization during the task. Independent t-tests on global alpha amplitude revealed no significant group differences for any MS class (all *p* > 0.05, Fig. 3A). In contrast to amplitude, independent t-tests demonstrated that ASD participants exhibited significantly lower PLV compared to TD participants across all five MS classes (Fig. 3A). Specifically, significant reductions were found for MS A (*p* = 0.011, Cohen’s *d* = 0.48, 95% CI = [0.12, 0.85]), MS B (*p* = 0.003, Cohen’s *d* = 0.56, 95% CI = [0.20, 0.94]), MS C (*p* = 0.001, Cohen’s *d* = 0.64, 95% CI = [0.27, 1.02]), MS D (*p* = 0.014, Cohen’s *d* = 0.46, 95% CI = [0.090, 0.82]), and MS E (*p* = 0.019, Cohen’s *d* = 0.44, 95% CI = [0.090, 0.82]). The number of MS-specific EEG time samples contributing to PLV estimation did not differ significantly between groups after FDR correction. No significant associations between PLV and time-sample count were observed, except for a positive association in MS E within ASD (*r* = 0.39, *p* = 0.019). After equalizing EEG time samples, PLV remained higher in TD than ASD for MS A, B, C, D and E (all *p* < 0.05) (Tables S4-6 in Additional file 1). All p-values were FDR-corrected.

To further determine whether the observed ASD-TD differences in MS reflect underlying patterns of phase synchronization, we analyzed the association between TD-referenced deviations in GFP and PLV within the ASD group. A significant negative correlation was observed for all five MS (MS A: *r* = -0.48, *p* = 1.27 × 10⁻⁴; MS B: *r* = -0.40, *p* = 1.80 × 10⁻³; MS C: *r* = -0.41, *p* = 1.43 × 10⁻³; MS D: *r* = -0.38, *p* = 3.41 × 10⁻³; MS E: *r* = -0.48, *p* = 1.16 × 10⁻⁴, FDR-corrected, Fig. 3B). This coupling was abolished after phase shuffling (all *p* > 0.05, Fig. 3B), confirming that intact phase structure is necessary.

### Identification of PLV modes across MS

Evaluation of candidate clustering solutions from K = 2 to K = 6 supported a two-mode solution (Fig. S3 in Additional file 1). Two distinct modes (K = 2) were identified as the optimal solution by k-means clustering on the PLV deviation. Mode 1 (*N* = 64) included 36 ASD and 28 TD participants, while mode 2 (*N* = 55) consisted of 22 ASD and 33 TD participants. Mode 2 exhibiting significantly higher PLV deviation scores than mode 1 across all MS (all *p* < 0.001, FDR-corrected, Fig. 3C). Specifically, a significant main effect of group was observed for MS C (*p* = 0.006, Cohen’s *d* = 0.60, 95% CI = (0.24, 0.99)), and a significant interaction was identified for MS A (*p* = 0.034, Cohen’s *d* = 0.80, 95% CI = (0.069, 1.64)). Post-hoc comparisons revealed significant differences between ASD and TD within mode 2 in both MS A (*p* = 0.026, Cohen’s *d* = 0.63, 95% CI = (0.065, 1.20)) and in MS C (*p* = 0.006, Cohen’s *d* = 0.78, 95% CI = (0.21, 1.35)).

### Mode comparison of MS-based PLV networks

**Fig. 4:**
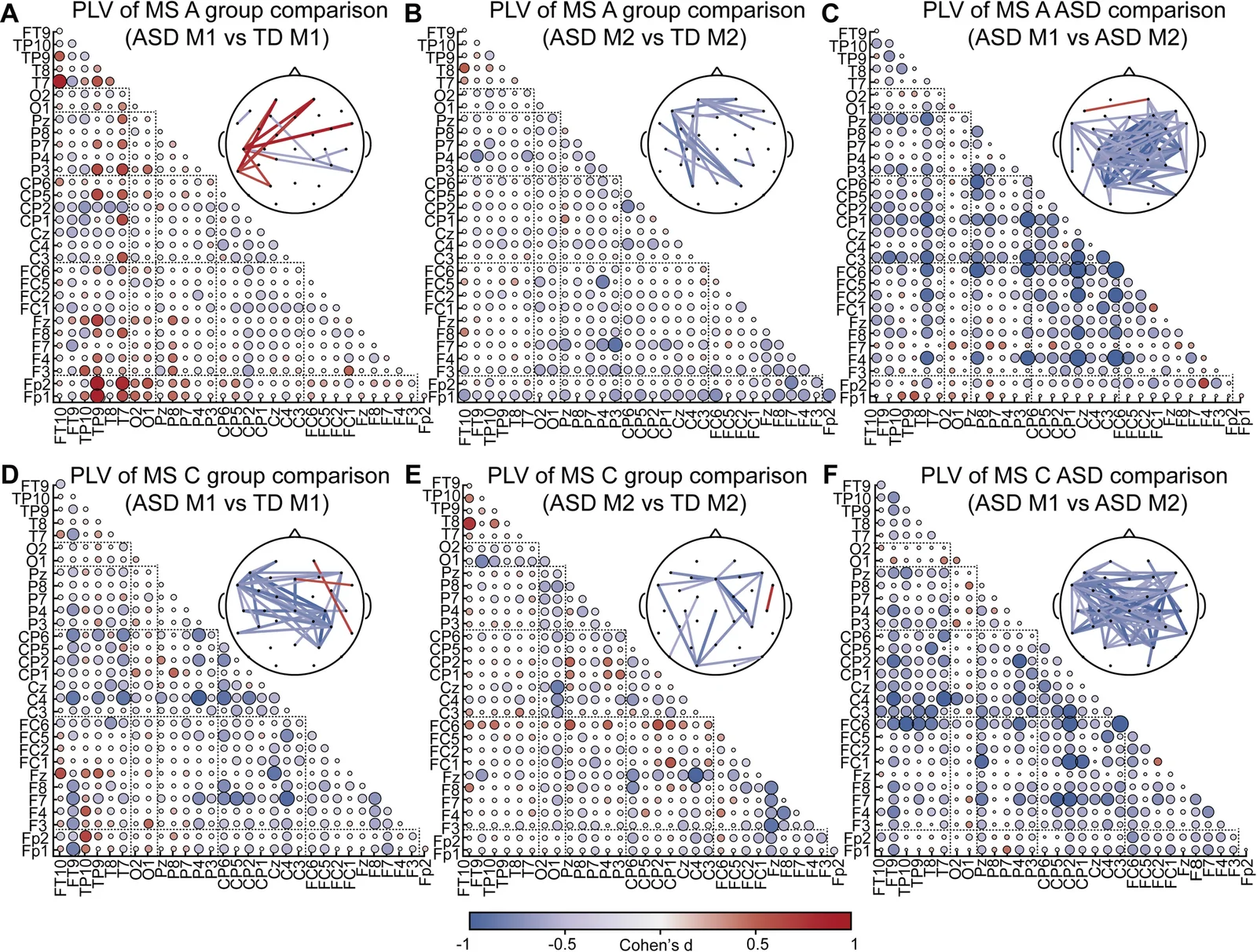
Network topology of phase synchronization differences. (**A-C**) Phase synchronization differences for MS A. Panels display the effect sizes of PLV differences for ASD mode 1 versus TD mode 1 (**A**), ASD mode 2 versus TD mode 2 (**B**), and ASD mode 1 versus mode 2 (**C**). (**D-F**) Phase synchronization differences for MS C. Panels display the corresponding comparisons: ASD mode 1 versus TD mode 1 (**D**), ASD mode 2 versus TD mode 2 (**E**), and ASD mode 1 versus mode 2 (**F**). In the main plots, the size of the circles represents the absolute value of Cohen’s *d*. The topographic insets at the top-right corner of each panel visualize edges with Cohen’s *d* greater than 0.6. ASD, autism spectrum disorder; MS, microstate; M1, mode 1; M2, mode 2; TD, typically developing

We visualized edgewise group differences in PLV using Cohen’s *d* values for MS A and C. This analysis was exploratory. For MS A, group differences showed several nominal effects, mainly in mode 1 (Fig. 4A). For MS C, group differences were more evident in mode 2 (Fig. 4E). To aid interpretation, electrodes were further grouped into six regions (Tables S7- 8 in Additional file 1). For MS A, mode 1 showed nominal ASD versus TD differences in within-prefrontal, frontal-parietal, and prefrontal-central coupling (Cohen’s *d* around − 0.73 to -0.51), and mode 2 showed a nominal difference in prefrontal-temporal coupling (Cohen’s *d* = 0.57), which did not survive FDR correction. The ASD mode comparison showed lower coupling in mode 1 than mode 2, with the strongest FDR-corrected effects for frontal-central (*p* = 0.007, Cohen’s *d* = 1.59), central-parietal (*p* < 0.001, Cohen’s *d* = 1.17), central-temporal (*p* < 0.001, Cohen’s *d* = 1.10), and within-central coupling (*p* < 0.001, Cohen’s *d* = 1.17).

For MS C, mode 1 showed nominal ASD versus TD differences in central-occipital and parietal-occipital coupling (Cohen’s *d* = -0.62 to -0.59), but none survived FDR correction. Mode 2 showed ASD versus TD differences in frontal-central and within-central coupling (both *p* = 0.002, *p* = 0.025, Cohen’s *d* = -0.80 and − 0.81, FDR-corrected), with additional nominal differences in central-temporal and within-frontal coupling. The ASD mode comparison showed lower coupling in mode 1 than mode 2, with the strongest FDR-corrected effects for frontal-central (*p* < 0.001, Cohen’s *d* = 1.40), central-temporal (*p* = 0.001, Cohen’s *d* = 1.21), central-parietal (*p* = 0.001, Cohen’s *d* = 1.13), and within-central coupling (*p* = 0.001, Cohen’s *d* = 1.11).

### Brain-behavior association

#### Associations between phase synchrony, autistic traits, and social gaze

From the eye-tracking data, SSPR did not differ between ASD and TD participants at the group level (*p* = 0.12). However, ASD participants showed lower valid looking (*p* = 0.036) and lower last-half SSPR (*p* = 0.034, Table S9). Within ASD, mode 1 showed lower SSPR than mode 2 (*p* = 0.009, Cohen’s *d* = 0.73, 95% CI = (0.19, 1.28), Fig. 3D). In contrast, SSPR did not differ between TD participants in mode 1 and mode 2 (*p* > 0.05). Within mode 1, ASD participants showed lower SSPR than TD participants, especially during the last half of the task (Table S10). Moreover, TD in mode 1 showed lower SRS-2 scores, although not significantly (*p* = 0.082).

To explore brain-behavior relationships, we examined associations between PLV deviation and demographic and behavioral measures. In mode 2, PLV deviation for MS A showed a significant positive correlation with SSPR (*r* = 0.49, *p* = 0.021, Fig. 3E), and PLV deviation for MS C showed a significant negative correlation with the social awareness subscale of the SRS-2 (*r* = -0.53, *p* = 0.019, Fig. 3F). No such associations reached significance in ASD mode 1 or in either TD mode (*p* > 0.05).

#### Moderation analyses linking phase synchrony, autistic traits, and social gaze

**Fig. 5:**
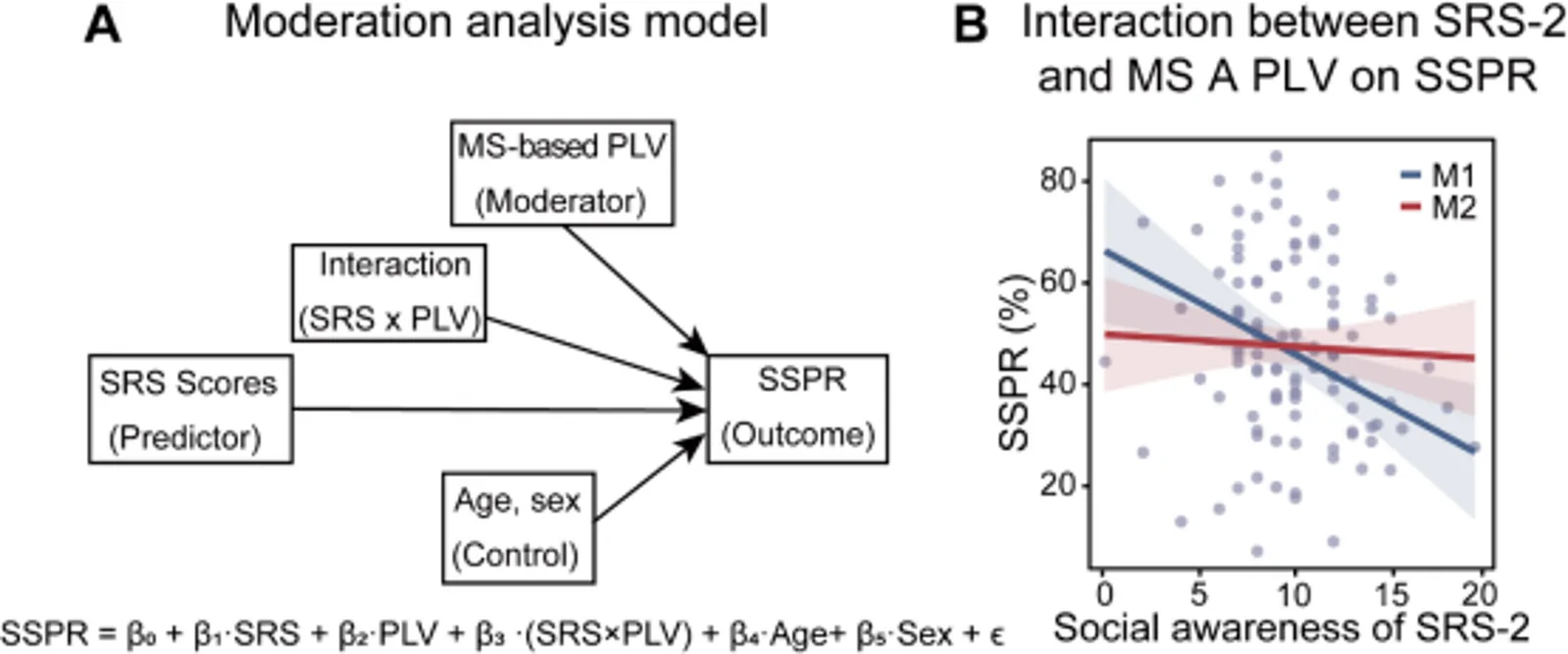
Moderation effect of alpha phase synchronization on the association between autistic traits and social preference. (**A**) Schematic illustration of the moderation model. (**B**) Interaction plot for the moderation model. The scatter plot illustrates the relationship between SRS-2 total score and SSPR as a function of PLV in MS A. Regression lines represent the simple slopes for individuals with low PLV (mode 1) and high PLV (mode 2). ASD, autism spectrum disorder; MS, microstate; PLV, phase locking value; M1, mode 1; M2, mode 2; SRS, Social Responsiveness Scale; SSPR, social scene preference ratio; TD, typically developing

A moderation analysis was performed to examine whether PLV modulated the relationship between SRS‑2 scores and SSPR. For MS A, the moderation model yielded a significant fit (F = 4.60, *p* = 0.001, R² = 0.18, adjusted R² = 0.18), with significant interaction between social awareness scores of the SRS-2 and PLV (*p* = 0.034). Simple slope analysis revealed that social awareness scores of the SRS-2 were negatively associated with SSPR among individuals with low PLV (i.e., mode 1, *p* = 0.007, Fig. 5B), whereas this association was not significant at high PLV levels (i.e., mode 2, *p* = 0.98, Fig. 5B). In contrast, although the overall model for MS C was significant (F = 4.13, *p =* 0.002, adjusted R²= 0.12), the interaction term was not significant (*p* = 0.923). No significant interactions were observed for other microstates (all *p* > 0.05). All p-values were FDR‑corrected.

### Sensitivity analysis

Sensitivity analysis using conventional measures yielded a consistent pattern, including better classification performance under the task condition than the resting-state condition and comparable PLV-based modes, although some effects were attenuated after correction for multiple comparisons (Full results are provided in Fig. S5 in Additional file 1).

To evaluate whether neural differences in mode 1 were driven by later gaze divergence, we recomputed PLV deviations using only the first-half EEG data. Estimates derived from the first-half did not differ from full-task estimates across the five microstates (Fig. S4 in Additional file 1).

## Discussion

The present study combined EEG and eye tracking to test whether variability in social orienting within ASD is associated with heterogeneous task-related neurodynamic modes. Four main findings emerged: (i) MS features extracted during the viewing condition differentiated ASD and TD groups more accurately than the same features extracted during resting state; (ii) group differences were primarily expressed in alpha phase synchronization, indexed by PLV, rather than alpha amplitude; (iii) clustering of MS-based PLV deviations identified two modes, and ASD in mode 1 showed lower SSPR than in mode 2; (iv) MS A phase synchronization moderated the relationship between autistic traits and SSPR within ASD mode 1. Together, these results suggest that ASD-related social deficits are associated with heterogeneous patterns of task-related neural coordination that become more apparent under social input and are better characterized by synchronization mechanisms than by amplitude differences alone.

### Task-based measures reveal ASD relevant dynamics more effectively than resting state measures

Task-based MS deviation features achieved substantially higher classification performance than resting state features. This pattern is consistent with the premise that social deficit related neural differences are state dependent, and can be underestimated when the brain is not required to process external social information. One likely explanation is that the viewing paradigm increases coordination demands across distributed networks that support attention, perceptual integration, and socially relevant cue processing [14, 42]. Even though the paradigm imposes low explicit task demands, it provides continuous, multimodal, time varying input that requires ongoing sampling and selection of socially meaningful information. In contrast, resting state may permit greater variability in internal mentation, arousal, and spontaneous strategies that can dilute group differences and increase within group heterogeneity [43, 44], thereby reducing discriminability. In addition, the paradigm places social and nonsocial dynamic information in direct competition under free-viewing conditions. The eye-tracking results show that ASD and TD participants did not differ significantly in overall SSPR, however, ASD participants showed lower SSPR during the last half of the task, suggesting a later shift away from social viewing. In this context, SSPR provides a behavioral anchor for interpreting neural dynamics, because it indexes the relative allocation of visual attention to social versus geometric stimuli. Therefore, the neural differences observed during viewing are likely to reflect the coordination demands required to sample, prioritize, and maintain engagement with socially meaningful information in the presence of visually salient nonsocial input.

The complexity results support this interpretation. TD participants showed a reduction in MS sequence complexity from rest to task, reflected in decreased MSE across several scales. This pattern suggests that the neural system of TD participants became more constrained and predictable under stimulus engagement, which is consistent with efficient entrainment to external structure and more stable allocation of network states during ongoing input processing [43, 45]. In contrast, participants with ASD maintained relatively high complexity across conditions. This absence of task induced constraint suggests reduced state modulation when processing dynamic stimuli [46, 47]. Mechanistically, this could reflect weaker social stimulus (i.e. social viewing) driven stabilization of network states [45], reduced context sensitivity [48], or altered regulation of arousal and attention [49] that prevents the typical transition from a more variable resting pattern to a more organized stimulus engaged pattern. From the perspective of ecological validity, the viewing condition appears to elicit precisely the type of state modulation that differentiates TD from ASD, thereby improving the signal to noise ratio for ASD relevant features.

### MS abnormalities are primarily expressed in phase synchronization rather than alpha amplitude

The second major finding was that task-based group differences were expressed in alpha phase synchronization across all MS classes. Consistent with the finding of the previous research that the key oscillatory properties of MS sequences are preferentially encoded by alpha phase rather than alpha amplitude [37]. PLV operationalizes the temporal stability of inter-signal phase relationships [50]. At the systems level, phase synchronization is widely used to index inter-regional connectivity, and synchrony-based dynamic links have been proposed as a plausible mechanism for large-scale functional integration [51]. This dissociation narrows the space of plausible electrophysiological mechanisms that can produce altered MS expression.

The widespread reduction of task-related PLV across MS is also broadly consistent with accounts of reduced long-range underconnectivity in ASD [52]. Earlier accounts have often emphasized a pattern of local overconnectivity together with reduced long-range or large-scale integration in ASD, although subsequent work suggests that this pattern may vary across development, task demands, frequency bands, and measurement modalities [53, 54]. Social orienting recruits parallel operations that combine multiple cues and engage attention, memory, and inferential processes in a dynamic context [55], and network integration has been argued to support adaptive behavior in complex naturalistic social settings [56]. In this context, alpha-band phase coupling may facilitate long-range information integration by temporally aligning segregated processing streams [57], and pre-stimulus alpha phase has been linked to gating feedforward information flow in early sensory processing [58]. Accordingly, reduced alpha synchronization would be expected to compromise the formation of stable, coordinated large-scale network states during viewing, which may plausibly contribute to ASD-related difficulties in social orienting.

This interpretation is supported by negative correlations between GFP and PLV across MS in ASD, which were abolished by phase shuffling, suggesting the observed ASD-related deviations in MS are embedded within a phase-organized regime. Converging evidence from ASD studies further indicates reduced alpha band phase synchronization relative to TD groups [59, 60], together supporting the interpretation that ASD-related alterations in MS expression are coupled to disruptions in large-scale phase coordination.

### PLV-based modes reveal neurodynamic heterogeneity in social orienting

Our clustering analysis revealed two distinct modes defined by MS based alpha band PLV deviations that were expressed in both ASD and TD. This pattern suggests that variability in large scale synchronization reflects a population level dimension. However, differences in social preference were not uniform across diagnostic groups. ASD participants in mode 1 showed lower SSPR than ASD in mode2, whereas no significant differences in SSPR were observed within TD. This pattern suggests that ASD in mode 1 was accompanied by reduced social orienting or reduced sustained engagement with social stimuli. The moderation effect provides a biologically informative link between synchronization and behavior. PLV during MS A in mode 1 altered the association between social deficit traits and SSPR, such that higher social awareness of SRS-2 scores predicted lower social viewing when synchronization was low, whereas this trait behavior association was absent when synchronization was higher. This pattern is consistent with the idea that phase synchronization indexes an available coordination resource that supports regulatory control of attention and constrains how strongly trait like vulnerabilities are expressed in observable behavior [57, 61]. When this coordination resource is limited, symptom severity more directly translates into reduced social orienting during viewing [62]. When it is relatively preserved, individuals may maintain social viewing despite elevated trait burden, consistent with buffering through top-down regulation and compensatory mechanisms [61, 63]. Alpha synchrony has been implicated in attentional gating and the coordination of long range communication [59], providing a plausible physiological substrate through which regulatory processes can stabilize engagement with socially meaningful cues.

Mode 2 was characterized by relatively preserved phase synchronization compared with mode 1, yet within ASD, MS specific PLV deviations scaled with autistic trait severity and social orienting, particularly for MS C. MS C has been linked to reflect the activity of the saliency network (SN) [21], a system believed to be dedicated to detecting and initiating responses to external and internal events that carry emotional or motivational salience [64, 65]. This association in current results suggests that the stability of the network state indexed by MS C is functionally relevant to social deficits in this context. It also implies that phase synchronization abnormalities are expressed in specific network states rather than uniformly across all MS, and that their magnitude scales with symptom severity of social deficit. Put differently, mode 2 may reflect a pathway in which core circuit level coordination deficits manifest as MS specific PLV reductions that contribute to social deficit. This observation is compatible with the idea that social deficit of ASD may depend on deficits in particular coordination regimes that are recruited during stimulus processing [66] and attention allocation [67, 68].

### Integrating PLV-based modes and MS specificity

Our results highlight MS A as a critical marker of neurodynamic heterogeneity, evidenced by its dominant contribution to SVM classification, distinct PLV deviations, and pivotal moderating role in the brain-behavior relationship. This convergence suggests that the coordination regime indexed by MS A may be especially relevant to the translation of autistic traits into social behavior. Specifically, for MS A which is linked to auditory and phonological processing [21, 69], mode 1 showed the most pronounced disruption in prefrontal-to-temporal coupling. These regions are critical nodes of the social brain, structurally connected by the arcuate fasciculus and functionally responsible for language comprehension and social-emotional processing [70–72]. The severe abnormality may reflect a failure to coordinate top-down control with sensory processing, potentially impeding the efficient prioritization of social signals. Notably, these findings should be interpreted cautiously, as PLV was estimated from a 32-channel montage, which limits source localization precision.

In stark contrast, mode 2 maintained relatively intact synchronization compared to mode 1, and the deficits were largely confined to local central regions in MS C. Spatially, MS C has been linked to both the SN [21] and the default mode network [73], which partially overlap with regions commonly implicated in the social brain [72, 74]. Functionally, MS C has been associated with processing personally significant information, self-reflection, and self-referential internal mentation [69], processes that are central to social cognition. This distinction suggests that the symptom linked profile of mode 2 does not reflect a widespread network collapse, but instead involves more specific disruptions in networks relevant to social processing.

### Limitations

Several limitations should be acknowledged. First, the weaker diagnostic separability at rest should not be taken to imply that resting state dynamics are uninformative, as they may capture clinically relevant variance beyond social impairment that was outside the scope of this study. Resting state data in children and adolescents are also more susceptible to uncontrolled factors such as compliance, mind wandering, and fluctuating arousal, which can obscure group differences. Second, although external validation of these data-driven modes is ideal, it was unfeasible in this study. The lack of comparable public datasets for our unique multimodal paradigm, combined with the high attrition rates and resource demands of pediatric EEG testing, precluded recruiting a second independent cohort. Third, the modes included both ASD and TD participants, which strengthens the dimensional interpretation but also indicates that the modes should not be treated as diagnostic categories. Future work should test whether mode membership predicts prognosis, treatment response, or developmental trajectories beyond diagnosis. Fourth, the design was cross sectional, which prevents causal inference about whether synchronization differences cause altered social orienting or reflect downstream consequences of development and experience. The age association observed motivates longitudinal studies to test whether individuals shift between modes over development. Fifth, it will also be important to examine whether similar dissociations between amplitude and synchronization hold in other frequency bands that contribute to social processing, including theta and beta bands, and whether cross frequency interactions better explain behavioral heterogeneity. Sixth, the present study did not include an independent diagnostic reassessment using gold-standard research instruments such as the Autism Diagnostic Observation Schedule or Autism Diagnostic Interview-Revised. Future studies should incorporate standardized diagnostic assessments to improve diagnostic characterization and facilitate comparison across samples. A further limitation concerns the incomplete characterization of cognitive functioning and medication or supplement use. Cognitive functioning/IQ was not systematically assessed, and the observed EEG effects should therefore be interpreted with caution, as unmeasured cognitive functioning and insufficiently characterized medication or supplement use may have contributed to inter-individual variability in neural measures. Finally, residual eye-movement contamination may not be completely excluded, despite ICA-based artifact correction. And participants with insufficient valid gaze data may represent a low-engagement subgroup, limiting generalizability to autistic individuals with minimal task engagement.

## Conclusion

The present findings support the use of ecologically meaningful stimulus driven paradigms paired with objective behavioral readouts when searching for mechanistic biomarkers of social deficit. Passive viewing with eye tracking provides a tractable bridge between resting state and interactive social tasks, which may be particularly suitable for autistic samples. Phase synchronization may provide more mechanistically informative biomarkers than alpha amplitude in this context. Additionally, the identification of modes that relate differently to symptoms and trait to behavior translation highlights the importance of considering neurodynamic heterogeneity when studying social orienting in ASD. These findings support the value of moving beyond group-average comparisons toward biologically informed, hypothesis-generating profiles, while emphasizing that such profiles should not be interpreted as discrete diagnostic subtypes without external validation.

## Supplementary Information

Below is the link to the electronic supplementary material.

Supplementary Material 1

## Data Availability

The anonymized dataset is available on reasonable request from the corresponding author.

## Abbreviations

ANOVA: Analysis of Variance
ASD: Autism Spectrum Disorder
AUC: Area Under the Receiver Operating Characteristic Curve
EEG: Electroencephalography
GFP: Global Field Power
MAD: Median Absolute Deviation
MS: Microstate
MSE: Multiscale Sample Entropy
PLV: Phase-Locking Value
ROC: Receiver Operating Characteristic
SRS: Social Responsiveness Scale
SRS-2: Social Responsiveness Scale, Second Edition
SSPR: Social Scene Preference Ratio
SVM: Support Vector Machine
TD: Typically Developing
