# Supplementary material for: Phase synchronization modes are associated with heterogeneous social orienting in children and adolescents with autism
Source: Mol Autism. 2026 Jul 9;17:39. doi: 10.1186/s13229-026-00729-w (PMC13632423; doi:10.1186/s13229-026-00729-w)
Supplement: Supplementary file 1 [file 13229_2026_729_MOESM1_ESM.docx]

**Figure S1.** Spatial similarity matrices between resting-state and task-related microstate templates for K = 3 to K = 6 solutions.

**Figure S2.** EEG microstate features comparison across groups and conditions. Significant main effects of group were observed for the duration (A, B, C, E), coverage (C), and occurrence (A, B, D, E) of MS (all *p* < 0.05, FDR-corrected). Regarding the main effect of condition, significant differences were limited to MS C in terms of occurrence and coverage (all *p* < 0.05, FDR-corrected). A significant interaction was observed specifically for the occurrence of MS C (*p* < 0.05). Post-hoc analysis for the occurrence of MS C revealed a significant difference between rest and task conditions specifically within the ASD group (*p* < 0.001), as well as a significant difference between ASD and TD groups during the task condition (*p* = 0.041). Raincloud plots showing individual deviation scores for EEG microstate duration, occurrence, and coverage across the five microstates. For each panel, distributions are displayed for ASD and TD under Rest and Task conditions. Positive values indicate higher-than-reference and negative values indicate lower-than-reference. ASD, autism spectrum disorder; MS, microstate; TD, typically developing; **p* < 0.05; *p* < 0.01; **p* < 0.001.

**Figure S3.** Evaluation of alternative cluster solutions. The five-dimensional PLV deviation profiles were reduced using principal component analysis before k-means clustering. Candidate solutions from K = 2 to K = 6 were evaluated using multiple internal validity indices. (A) Silhouette coefficient, with higher values indicating better within-cluster cohesion and between-cluster separation. (B) Calinski-Harabasz index, with higher values indicating better-defined clusters. (C) Gap statistic. The one-standard-error criterion supported the more parsimonious two-mode solution. (D) Davies-Bouldin index, with lower values indicating better compactness and separation. (E) Clustering stability quantified by the mean pairwise adjusted Rand index across 500 k-means repetitions. Error bars indicate standard deviations. (F) PCA scatter plot of the final two-mode solution. Points are displayed according to diagnostic group and PLV-based neural mode. ASD, autism spectrum disorder; TD, typically developing; ARI, adjusted Rand index; M1, mode 1; M2, mode 2.

**Figure S4.** Gaze behavior and robustness of PLV deviation estimates.

(A) SSPR in the full task, first half, and last half for ASD and TD groups. (B) Association between first-half and last-half SSPR across participants. (C) Comparison between full-task and first-half PLV deviation within mode 1 across microstates A–E. ASD, autism spectrum disorder; MS, microstate; PLV, phase-locking value; SSPR, social scene preference ratio; TD, typically developing. * p < 0.05, *** p < 0.001; ns, not significant.

**Figure S5.** Sensitivity analysis based on group-level measures without TD-referenced individualized deviation scores. (A) ROC curves comparing the classification performance of resting-state and task-based MS features in distinguishing ASD from TD participants. The task condition showed a higher AUC than the resting-state condition. (B) Confusion matrices from the 5-fold cross-validated SVM classifiers for resting-state and task-based MS features. (C) Data-driven PLV modes derived from MS-based PLV features. The two modes showed significant differences across the five MS. Boxplots show the distribution of standardized PLV values for MS A–E, separately for ASD and TD participants within each PLV mode. (D) Differences in SSPR between the two PLV modes in ASD and TD participants. ASD participants in M2 showed higher SSPR than those in M1, whereas this difference was not significant in TD participants. (E) Correlation between PLV of MS C and the social awareness subscale of the SRS-2 in ASD participants within M2. (F) Interaction plot for the moderation model. The scatter plot illustrates the relationship between SRS-2 social awareness and SSPR as a function of PLV in MS A. Regression lines represent the simple slopes for individuals with low PLV (M1) and high PLV (M2). ASD, autism spectrum disorder; AUC, area under the curve; MS, microstate; ns, no significance; PLV, phase locking value; M1, mode 1; M2, mode 2; SRS-2, Social Responsiveness Scale, Second Edition; SSPR, social scene preference ratio; TD, typically developing; *p < 0.05; **p < 0.01; ***p < 0.001.

**Table S1.** Validation indices for different microstate solutions

| **Number of MS** | **Resting GEV (%)** | **Task GEV (%)** | **Mean rest-task spatial similarity** |
| --- | --- | --- | --- |
| K = 3 | 68.03 | 67.98 | 0.95 |
| K = 4 | 71.48 | 71.45 | 0.84 |
| K = 5 | 73.6 | 73.74 | 0.97 |
| K = 6 | 75.19 | 75.29 | 0.84 |

GEV, global explained variance; MS, microstate.

**Table S2.** Differences in MSE between ASD and TD within each condition

|  | **Rest** | | |  | **Task** | | |
| --- | --- | --- | --- | --- | --- | --- | --- |
| **Scale** | ***p*** | ***p.adj*** | **Cohen’s *d* [95% CI]** |  | ***p*** | ***p.adj*** | **Cohen’s *d* [95% CI]** |
| 1 | 0.78 | 0.96 | -0.045 [-0.26, 0.37] | | 0.015 | 0.022 | 0.45 [0.10, 0.80] |
| 2 | 0.99 | 0.99 | 0.001 [-0.31, 0.32] | | 0.013 | 0.022 | 0.46 [0.12, 0.80] |
| 3 | 0.72 | 0.96 | -0.056 [-0.37, 0.26] | | 0.013 | 0.022 | 0.46 [0.12, 0.82] |
| 4 | 0.86 | 0.96 | -0.027 [-0.33, 0.30] | | 0.003 | 0.013 | 0.56 [0.25, 0.86] |
| 5 | 0.49 | 0.96 | -0.11 [-0.41, 0.21] | | 0.005 | 0.016 | 0.52 [0.21, 0.82] |
| 6 | 0.18 | 0.61 | -0.21 [-0.50, 0.10] | | 0.007 | 0.018 | 0.50 [0.19, 0.78] |
| 7 | 0.030 | 0.27 | -0.35 [-0.64, -0.047] | | <0.001 | 0.003 | 0.67 [0.35, 1.0] |
| 8 | 0.78 | 0.96 | 0.043 [-0.26, 0.35] | | 0.17 | 0.21 | 0.25 [-0.090, 0.61] |
| 9 | 0.54 | 0.96 | 0.096 [-0.20, 0.40] | | 0.89 | 0.89 | 0.025 [-0.34, 0.39] |
| 10 | 0.053 | 0.27 | -0.30 [-0.63, -0.007] | | 0.29 | 0.32 | 0.19 [-0.17, 0.53] |

Cohen’s d was calculated as ASD-TD. ASD, autism spectrum disorder; TD, typically developing.

**Table S3.** Differences in MSE between resting-state and task conditions within each group

|  | **ASD** | | |  | **TD** | | |
| --- | --- | --- | --- | --- | --- | --- | --- |
| **Scale** | ***p*** | ***p.adj*** | **Cohen’s *d* [95% CI]** |  | ***p*** | ***p.adj*** | **Cohen’s *d* [95% CI]** |
| 1 | 0.45 | 0.52 | 0.13 [-0.20, 0.44] | | 0.091 | 0.11 | -0.31 [-0.66, 0.034] |
| 2 | 0.40 | 0.52 | 0.14 [-0.18, 0.46] | | 0.067 | 0.11 | -0.33 [-0.66, 0.004] |
| 3 | 0.17 | 0.52 | 0.22 [-0.090, 0.55] | | 0.084 | 0.11 | -0.31 [-0.66, 0.032] |
| 4 | 0.23 | 0.52 | 0.19 [-0.12, 0.48] | | 0.020 | 0.049 | -0.43 [-0.76, -0.092] |
| 5 | 0.31 | 0.52 | 0.16 [-0.15, 0.45] | | 0.010 | 0.032 | -0.48 [-0.81, -0.16] |
| 6 | 0.28 | 0.52 | 0.17 [-0.14, 0.44] | | 0.005 | 0.025 | -0.52 [-0.83, -0.20] |
| 7 | 0.030 | 0.30 | 0.35 [0.035, 0.66] | | <0.001 | 0.003 | -0.67 [-1.01, -0.34] |
| 8 | 0.81 | 0.81 | 0.035 [-0.28, 0.37] | | 0.33 | 0.36 | -0.18 [-0.52, 0.18] |
| 9 | 0.46 | 0.52 | -0.12 [-0.46, 0.21] | | 0.76 | 0.76 | -0.055 [-0.41, 0.29] |
| 10 | 0.32 | 0.52 | 0.17 [-0.16, 0.49] | | 0.079 | 0.11 | -0.32 [-0.66, 0.022] |

Cohen’s d was calculated as Rest-Task. ASD, autism spectrum disorder; TD, typically developing.

**Table S4.** Group differences in the number of microstate-specific EEG time samples contributing to PLV estimation.

| **Microstate** | **t(df)** | ***p*** | ***p.adj*** | **Cohen's *d* [95% CI]** |
| --- | --- | --- | --- | --- |
| A | 1.44 (117) | 0.15 | 0.76 | -0.26 [-0.63, 0.097] |
| B | 0.18 (117) | 0.86 | 0.97 | -0.033 [-0.39, 0.33] |
| C | -0.70 (117) | 0.49 | 0.81 | 0.13 [-0.23, 0.49] |
| D | -0.039 (117) | 0.97 | 0.97 | 0.007 [-0.35, 0.37] |
| E | -0.76 (117) | 0.45 | 0.81 | 0.14 [-0.22, 0.50] |

Positive t and Cohen’s d indicate TD > ASD. ASD, autism spectrum disorder; TD, typically developing.

**Table S5.** Associations between PLV and the number of microstate-specific EEG time samples.

|  | **ASD** | | |  | **TD** | | |
| --- | --- | --- | --- | --- | --- | --- | --- |
| **Microstate** | ***r*** | ***p*** | ***p.adj*** |  | ***r*** | ***p*** | ***p.adj*** |
| A | -0.084 | 0.53 | 0.65 |  | 0.22 | 0.092 | 0.42 |
| B | -0.069 | 0.61 | 0.65 |  | -0.18 | 0.17 | 0.42 |
| C | 0.21 | 0.11 | 0.26 |  | 0.075 | 0.57 | 0.57 |
| D | -0.060 | 0.65 | 0.65 |  | -0.088 | 0.53 | 0.57 |
| E | 0.38 | 0.004 | 0.019 |  | -0.092 | 0.48 | 0.57 |

ASD, autism spectrum disorder; TD, typically developing.

**Table S6.** Group differences in PLV with equal EEG time samples after 1,000 subsampling iterations

| **Microstate** | **t(df)** | ***p*** | ***p.adj*** | **Cohen's *d* [95% CI]** |
| --- | --- | --- | --- | --- |
| A | -2.59 (117) | 0.011 | 0.017 | 0.48 [0.11, 0.84] |
| B | -3.08 (117) | 0.003 | 0.007 | 0.56 [0.20, 0.93] |
| C | -3.47 (117) | <0.001 | 0.004 | 0.64 [0.27, 1.0] |
| D | -2.50 (117) | 0.014 | 0.017 | 0.46 [0.094, 0.82] |
| E | -2.39 (117) | 0.019 | 0.019 | 0.44 [0.074, 0.80] |

Positive t and Cohen’s d indicate TD > ASD. ASD, autism spectrum disorder; TD, typically developing.

**Table S7.** Region pair level PLV group differences for microstate A (*p* < 0.05)

| **Contrast** | **Region pair** | **Cohen’s *d*** | ***p*** | ***p.adj*** |
| --- | --- | --- | --- | --- |
| ASD M1 vs TD M1 | Prefrontal-Prefrontal | -0.73 | 0.012 | 0.25 |
| ASD M1 vs TD M1 | Frontal-Parietal | -0.54 | 0.042 | 0.28 |
| ASD M1 vs TD M1 | Prefrontal-Central | -0.51 | 0.048 | 0.28 |
| ASD M2 vs TD M2 | Prefrontal-Temporal | 0.57 | 0.033 | 0.40 |
| ASD M1 vs ASD M2 | Frontal-Central | 0.99 | <0.001 | 0.007 |
| ASD M1 vs ASD M2 | Frontal-Temporal | 0.70 | 0.014 | 0.12 |
| ASD M1 vs ASD M2 | Central-Temporal | 0.66 | 0.017 | 0.12 |
| ASD M1 vs ASD M2 | Central-Central | 0.60 | 0.031 | 0.13 |
| ASD M1 vs ASD M2 | Frontal-Frontal | 0.62 | 0.032 | 0.13 |
| ASD M1 vs ASD M2 | Frontal-Central | 0.99 | 0.000 | 0.007 |
| ASD M1 vs ASD M2 | Frontal-Temporal | 0.70 | 0.014 | 0.12 |
| ASD M1 vs ASD M2 | Central-Temporal | 0.66 | 0.017 | 0.12 |
| ASD M1 vs ASD M2 | Central-Central | 0.60 | 0.031 | 0.13 |
| ASD M1 vs ASD M2 | Frontal-Frontal | 0.62 | 0.032 | 0.13 |
| ASD M1 vs ASD M2 | Frontal-Central | 0.99 | 0.000 | 0.007 |
| ASD M1 vs ASD M2 | Frontal-Temporal | 0.70 | 0.014 | 0.12 |
| ASD M1 vs ASD M2 | Central-Temporal | 0.66 | 0.017 | 0.12 |
| ASD M1 vs ASD M2 | Central-Central | 0.60 | 0.031 | 0.13 |
| ASD M1 vs ASD M2 | Frontal-Frontal | 0.62 | 0.032 | 0.13 |
| ASD M1 vs ASD M2 | Frontal-Central | 1.59 | <0.001 | <0.001 |
| ASD M1 vs ASD M2 | Central-Parietal | 1.17 | <0.001 | <0.001 |
| ASD M1 vs ASD M2 | Central-Temporal | 1.10 | <0.001 | <0.001 |
| ASD M1 vs ASD M2 | Central-Central | 1.17 | <0.001 | <0.001 |
| ASD M1 vs ASD M2 | Parietal-Temporal | 0.89 | <0.001 | 0.002 |
| ASD M1 vs ASD M2 | Frontal-Temporal | 0.75 | 0.008 | 0.027 |
| ASD M1 vs ASD M2 | Frontal-Frontal | 0.69 | 0.013 | 0.038 |
| ASD M1 vs ASD M2 | Parietal-Parietal | 0.66 | 0.016 | 0.042 |
| ASD M1 vs ASD M2 | Frontal-Parietal | 0.67 | 0.022 | 0.052 |

ASD, autism spectrum disorder; TD, typically developing.

**Table S8** Region pair level PLV group differences for microstate C (*p* < 0.05)

| **Comparison** | **Region pair** | **Cohen’s *d*** | ***p*** | ***p.adj*** |
| --- | --- | --- | --- | --- |
| ASD M1 vs TD M1 | Central-Occipital | -0.62 | 0.033 | 0.27 |
| ASD M1 vs TD M1 | Parietal-Occipital | -0.59 | 0.045 | 0.27 |
| ASD M2 vs TD M2 | Frontal-Central | -0.80 | 0.002 | 0.025 |
| ASD M2 vs TD M2 | Central-Central | -0.81 | 0.002 | 0.025 |
| ASD M2 vs TD M2 | Central-Temporal | -0.68 | 0.010 | 0.070 |
| ASD M2 vs TD M2 | Frontal-Frontal | -0.55 | 0.037 | 0.19 |
| ASD M1 vs ASD M2 | Central-Central | 0.71 | 0.017 | 0.33 |
| ASD M1 vs ASD M2 | Frontal-Central | 0.56 | 0.035 | 0.33 |
| ASD M1 vs ASD M2 | Central-Temporal | 0.56 | 0.048 | 0.33 |
| ASD M1 vs ASD M2 | Central-Central | 0.71 | 0.017 | 0.33 |
| ASD M1 vs ASD M2 | Frontal-Central | 0.56 | 0.035 | 0.33 |
| ASD M1 vs ASD M2 | Central-Temporal | 0.56 | 0.048 | 0.33 |
| ASD M1 vs ASD M2 | Central-Central | 0.71 | 0.017 | 0.33 |
| ASD M1 vs ASD M2 | Frontal-Central | 0.56 | 0.035 | 0.33 |
| ASD M1 vs ASD M2 | Central-Temporal | 0.56 | 0.048 | 0.33 |
| ASD M1 vs ASD M2 | Frontal-Central | 1.40 | <0.001 | <0.000 |
| ASD M1 vs ASD M2 | Central-Temporal | 1.21 | <0.001 | 0.001 |
| ASD M1 vs ASD M2 | Central-Parietal | 1.13 | <0.001 | 0.001 |
| ASD M1 vs ASD M2 | Central-Central | 1.11 | <0.001 | 0.001 |
| ASD M1 vs ASD M2 | Parietal-Temporal | 0.95 | 0.001 | 0.003 |
| ASD M1 vs ASD M2 | Prefrontal-Frontal | 0.89 | 0.001 | 0.004 |
| ASD M1 vs ASD M2 | Frontal-Frontal | 0.92 | 0.001 | 0.004 |
| ASD M1 vs ASD M2 | Frontal-Parietal | 0.71 | 0.010 | 0.025 |
| ASD M1 vs ASD M2 | Frontal-Temporal | 0.79 | 0.011 | 0.025 |
| ASD M1 vs ASD M2 | Temporal-Temporal | 0.68 | 0.021 | 0.044 |

ASD, autism spectrum disorder; TD, typically developing.

**Table S9.** Gaze data quality and social preference during the passive viewing task

| **Measure** | **ASD, n=58, mean (SD)** | **TD, n=61, mean (SD)** | ***p*** | **Cohen’s *d* [95% CI]** |
| --- | --- | --- | --- | --- |
| Valid looking (%) | 88.80 (14.72) | 93.33 (7.48) | 0.036 | -0.39 [-0.76, -0.03] |
| Overall SSPR (%) | 44.40 (12.10) | 49.17 (19.81) | 0.12 | -0.29 [-0.65, 0.07] |
| SSPR, first-half (%) | 47.81 (17.00) | 50.06 (19.96) | 0.52 | -0.12 [-0.49, 0.24] |
| SSPR, last-half (%) | 40.26 (16.83) | 48.20 (22.34) | 0.034 | -0.40 [-0.77, -0.03] |

Cohen’s d was calculated as ASD-TD. ASD, autism spectrum disorder; SSPR, social scene preference ratio; TD, typically developing.

**Table S10.** Gaze data quality and social preference within different modes during the passive viewing task

|  | **Mode 1** | | | |  | **Mode 2** | | | |
| --- | --- | --- | --- | --- | --- | --- | --- | --- | --- |
| **Measure** | **ASD, n=36, mean (SD)** | **TD, n=28, mean (SD)** | ***p*** | **Cohen’s *d* [95% CI]** |  | **ASD, n=22, mean (SD)** | **TD, n=33, mean (SD)** | ***p*** | **Cohen’s *d* [95% CI]** |
| Valid looking (%) | 85.84 (16.21) | 91.67 (9.72) | 0.10 | -0.43 [-0.93, 0.08] |  | 93.60 (10.58) | 94.74 (4.55) | 0.59 | -0.15 [-0.70, 0.39] |
| Overall SSPR (%) | 41.20 (11.10) | 51.21 (20.16) | 0.014 | -0.64 [-1.14, -0.13] |  | 49.63 (12.07) | 47.45 (19.66) | 0.64 | 0.13 [-0.41, 0.67] |
| SSPR, first-half (%) | 44.42 (16.02) | 52.17 (20.11) | 0.096 | -0.43 [-0.94, 0.07] |  | 53.30 (17.48) | 48.28 (19.97) | 0.35 | 0.26 [-0.29, 0.81] |
| SSPR, last-half (%) | 37.05 (16.89) | 50.23 (22.74) | 0.011 | -0.67 [-1.18, -0.15] |  | 45.45 (15.77) | 46.48 (22.19) | 0.85 | -0.05 [-0.60, 0.50] |

Cohen’s d was calculated as ASD-TD. ASD, autism spectrum disorder; SSPR, social scene preference ratio; TD, typically developing.
